# Prognostic significance of lymphovascular space invasion in early-stage low-grade endometrioid endometrial cancer: a fifteen-year retrospective Chinese cohort study

**DOI:** 10.1186/s12957-024-03483-6

**Published:** 2024-07-30

**Authors:** Bowen Sun, Xiaobo Zhang, Yangyang Dong, Xingchen Li, Xiao Yang, Lijun Zhao, Jianliu Wang, Yuan Cheng

**Affiliations:** 1 Department of Obstetrics and Gynecology, Peking University People’s Hospital, Beijing, 100044 China; 2 Department of Pathology, Peking University People’s Hospital, Beijing, 100044 China

**Keywords:** LVSI, Endometrial cancer, Early-stage, Low-grade, Prognosis

## Abstract

**Objective:**

In 2016, the ESMO-ESGO-ESTRO consensus included LVSI (Lymph-vascular space invasion, LVSI) status as a risk stratification factor for stage I endometrioid endometrial cancer (EEC) patients and as one of the indications for adjuvant therapy. Furthermore, LVSI is included in the new FIGO staging of endometrial cancer (EC) in 2023. However, the data contribution of the Chinese population in this regard is limited. The present study aimed to further comfirm the influence of LVSI on the prognosis of early-stage low-grade EEC in a fifteen-year retrospective Chinese cohort study.

**Methods:**

This retrospective analysis cohort included 702 EEC patients who underwent TAH/BSO surgery, total abdominal hysterectomy, bilateral salpingooophorectomy in Peking University People’s Hospital from 2006 to 2020. Patients were stratified based on LVSI expression status as: LVSI negative group and LVSI positive group. Clinical outcome measures related to LVSI, assessed with a univariate and multivariate Cox proportional hazards regression model.

**Results:**

702 EEC patients with stage I and grade 1–2 were analyzed. 58 patients (8.3%) were LVSI-positive and 14 patients (2.0%) was relapse. Recurrence rates in LVSI-negative and LVSI-positive were 1.6% and 6.9%, respectively. 5-year disease-free survival (DFS) rate in LVSI-negative and LVSI-positive were 98.4% and 93.1%, respectively. These rates for 5-year overall (OS) survival in LVSI-negative were 98.9% while it was 94.8% in LVSI-positive. Multivariate analysis showed that LVSI is an independent risk factor for 5-year DFS (HR = 4.60, *p* = 0.010). LVSI has a similar result for 5-year OS(HR = 4.39, *p* = 0.028).

**Conclusions:**

LVSI is an independent predictor of relapse and poor prognosis in early-stage low-grade endometrioid endometrial cancer in the Chinese cohort.

## Introduction

Endometrial cancer (EC) is the fourth most common gynecologic malignant tumor with over 66,200 estimated new cases at 2023 in the United States, while being the sixth most common gynecological cancer worldwide [1]. From 2000 to 2015, the incidence and mortality of carcinoma of corpus uteri have increased among Chinese women [2]. In general, most patients with endometrial cancer have good prognosis, excluding some special pathological types, such as serous endometrial carcinoma, clear cell carcinoma, etc. Endometrioid endometrial cancer (EEC) accounts for 80% of all endometrial cancers, and a large proportion of patients are at early-stage, low-grade more than 60% [3, 4]. However, the prognosis of early-stage, low-grade EC patients is still affected by a series of risk factors, but the research data are still few, and the relevant data of the Chinese population cohort are even less.

Lymph-vascular space invasion (LVSI) is described as the occurrence of cancer cells in the lymphatic and circulatory systems’ vessels. According to some studies, LVSI is an independent risk factor for lymph node metastasis (LNM) in EC and is considered a predictor of LNM [5–8]. Previous research has shown that LNM is predictive in preoperative assessment and prognosis [9–11]. In recent years, the prognostic value of LVSI in patients with endometrial cancer without lymph node metastasis or confined to the uterus has been gradually explored. Some studies report that LVSI significantly reduces overall survival (OS) and disease-free survival (DFS) in EC patients, particularly in early-stage EC [12–17]. In contrast, other studies suggest that LVSI does not significantly impact survival outcomes [18–22]. Among LNM-negative patients, clinical outcomes are similar regardless of LVSI presence [20].

According to the 2023 International Federation of Gynaecology and Obstetrics (FIGO) staging system, LVSI is included in the standards of stage I with or without focal LVSI and Stage II associated with substantial LVSI distribution [23]. The revision of 2023 FIGO staging confirmed the important role of LVSI in the prognosis of stage I and stage II patients [24, 25]. However, the impact of LVSI on the prognosis of l early-stage, low-grade endometrial cancer is still lack of more evidence, especially the cohort data of more than 10 years in Chinese population. This study aims to determine whether LVSI impacts the prognosis of patients with low-grade and early-stage endometrioid endometrial cancer, mainly gathered in women with stage I (IA and IB), Grade 1/2. Our analyses rely on clinical participant data who underwent surgery and their follow-up data to associate LVSI with EC prognosis.

## Materials and methods

**Fig. 1:**
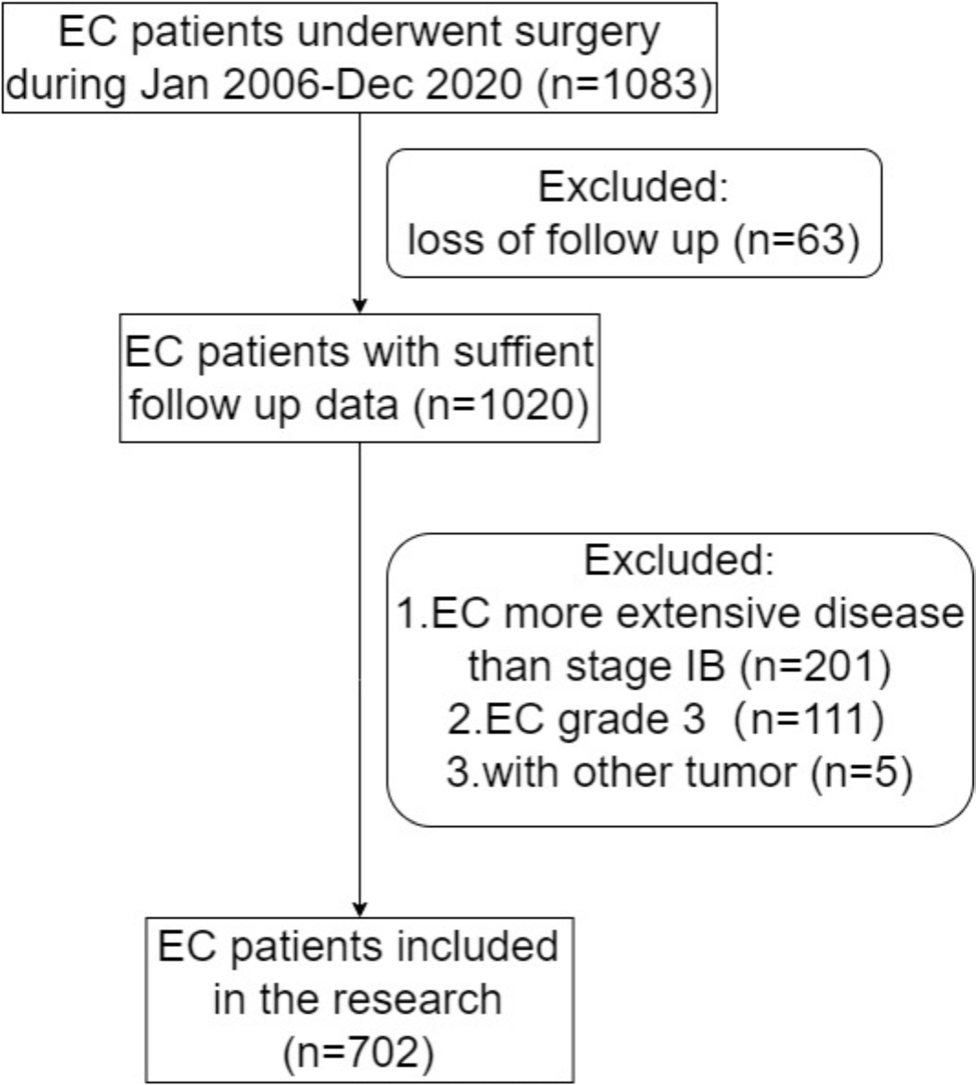
Flowchart describing participant selection in the study. ^a^EC: endometrial cancer

After obtaining authorization from the Ethics Committee of Peking University People’s Hospital (Institutional Review Board Approval Number: 2019PHB031-01, 8 March 2019), We analyzed their basic, hospital, and follow-up information. The population of this study included patients with endometrioid histology grade 1 or 2 and EC at stage IA or IB, as established by their final pathology reports. We first excluded patients with incomplete medical records (*n* = 63). We then excluded patients with tumors more advanced than stage IB after surgical staging (*n* = 201) and grade 3 diseases (*n* = 111). Women with other tumors were also excluded (*n* = 5) **(**Fig. 1**)**.

Gynecological oncologists performed total hysterectomy, bilateral salpingo-oophorectomy, selective bilateral pelvic and para-aortic lymphadenectomy, and pelvic washing on all eligible patients. We collected data on demographic, clinical, and surgical variables and evaluated them in this study. This included each patient’s age at first diagnosis, menopausal status, recurrence, time to recurrence, adjuvant treatment, follow-up duration, and survival time. We reviewed all original pathology reports and extracted pathological information. Experienced independent gynecological pathologists reviewed the pathology slides. We discussed controversial cases in expert meetings to reach a final decision. We assessed the grade, primary diameter of the tumor, and LVSI. All cases were staged according to the 2009 FIGO staging system [23]. Patients treated before 2009 were restaged based on clinicopathological data. Tumor histology and grade were assessed according to the World Health Organisation (WHO) classification system and FIGO criteria [26]. LVSI was diagnosed by identifying adenocarcinoma of any grade in endothelium-lined channels of uterine specimens at surgery [27]. We followed up with all patients after surgery through outpatient visits or phone calls. Telephone follow-up was conducted for patients regularly consulted at other hospitals. We collected information on symptoms and results from pelvic ultrasound, computed tomography (CT), or magnetic resonance imaging (MRI). We recorded any recurrences and deaths during the follow-up. If telephone follow-up revealed abnormal findings, patients were asked to undergo detailed examinations at our center. A diagnosis of relapse was made accordingly. Reasons for loss to follow-up included death from EC or other causes, loss to follow-up, and reaching the end of follow-up (October 31, 2022). Overall survival (OS) is the time from surgery to death from any cause, including a tumor. DFS was the time from surgery to confirmed relapse of EC. In survival analysis, patients without an event at their last follow-up were censored.

We compared clinicopathological factors between LVSI-negative and LVSI-positive patients. We used the student t-test and χ2 test for comparing continuous and categorical variables, respectively. Kaplan-Meier survival analyses (log-rank tests) and univariate Cox regressions were used to identify potential prognostic indicators. Multivariate Cox regression was used to analyze the survival influence of LVSI. In these regressions, we included factors with *p* < 0.05 in univariate analyses, in addition to LVSI. We examined the proportional hazard hypothesis with time-dependent covariates in all Cox regression models. Stepwise Cox regressions helped eliminate multicollinearity between variables, selecting important survival predictors for patients. Variables included were those with *p* < 0.05 in univariate Cox regressions. The analysis method was forward-biased: conditional, with an entry criterion for variables at *p* < 0.05. We conducted statistical analyses using SPSS software (version 27.0; IBM Corporation, USA), setting a significance level at *p* < 0.05.

## Results

We enrolled a total of 702 eligible EC patients in this study. Of the 702 cases, the study population had a median age of 55 years (range 24–83) and the cases were followed up for a median of 62.4 months (range 0.4–195). Fifty-eight patients (8.3%) present LVSI positve. All patients underwent lymphadenectomy of both pelvic and para-aortic regions. After surgery, 235 patients (33.5%) received adjuvant therapy.

We displayed demographic and clinicopathological characteristics of low-grade and early-stage EEC women, based on LVSI status (Table 1**).** Patients testing positive for LVSI were more likely to have more extensive myometrial invasion (*p* < 0.001) and increased tumor size (*p* < 0.001) compared to those testing negative for LVSI. Compared to LVSI-negative patients, those who were LVSI-positive were more likely to receive adjuvant treatment (197/644 vs. 38/58, respectively, *p* < 0.001) and experience relapse during follow-up (10/644 vs. 4/58, respectively, *p* = 0.022).

**Table 1:** Demographic and clinicopathological characteristics of low-grade and early stage endometrioid endometrial cancer patients (*n* = 702) according to LVSI status

Characteristics	Total	LVSI(-) *n* = 644	LVSI(+) *n* = 58	*p* value
Age(years, median)	55.23 ± 0.35	54.79 ± 0.36	60.10 ± 1.10	0.126
< 60	484(68.9)	452(70.2)	32(55.2)	
≥ 60	218(31.1)	192(29.8)	26(44.8)	
BMI				0.485
< 24	216(30.8)	197(30.6)	19(32.8)	
≥ 24 and < 28	243(34.6)	220(34.2)	23(34.6)	
≥ 28	243(34.6)	227(35.2)	16(27.6)	
Menopausal status				0.013
Pre-menopausal	264(37.6)	251(39.0)	13(22.4)	
Post-menopausal	438(62.4)	393(61.0)	45(77.6)	
Grade				0.049
G1	341(48.6)	320(49.7)	21(36.2)	
G2	361(51.4)	324(50.3)	37(63.8)	
Depth of MI				< 0.001
≤ 50%	597(85.0)	563(87.4)	34(58.6)	
> 50%	105(15.0)	81(12.6)	24(41.4)	
PTD (cm), median				< 0.001
≤ 20 mm	363(51.7)	347(53.9)	16(27.6)	
> 20 mm	339(48.3)	297(46.1)	42(72.4)	
Adjuvant treatment				< 0.001
No	467(66.5)	447(69.4)	20(34.5)	
Yes	235(33.5)	197(30.6)	38(65.5)	
Relapse				0.022
No	688(98.0)	634(98.4)	54(93.1)	
Yes	14(2.0)	10(1.6)	4(6.9)	

LVSI, lymphovascular space invasion; MI, myometrial invasion; PTD, primary tumor diameter

**Fig. 2:**
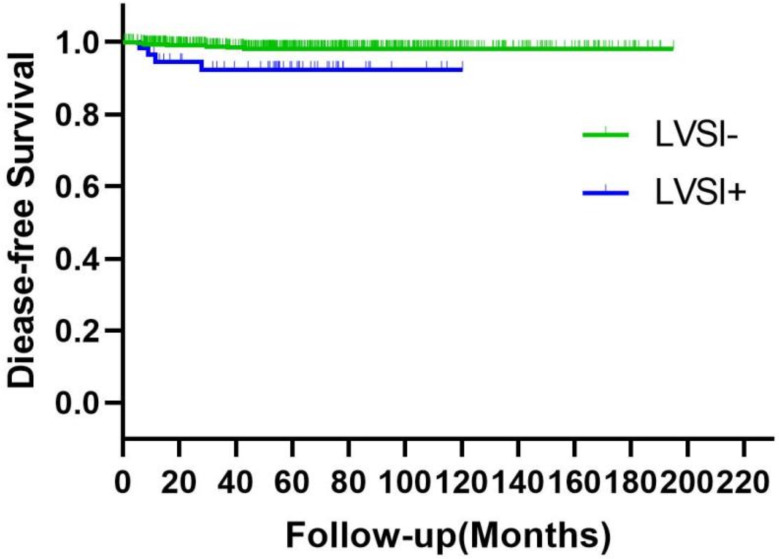
DFS of patients with negative and positive LVSI. ^a^DFS: disease-free survival; LVSI: lymphovascular space invasion

**Fig. 3:**
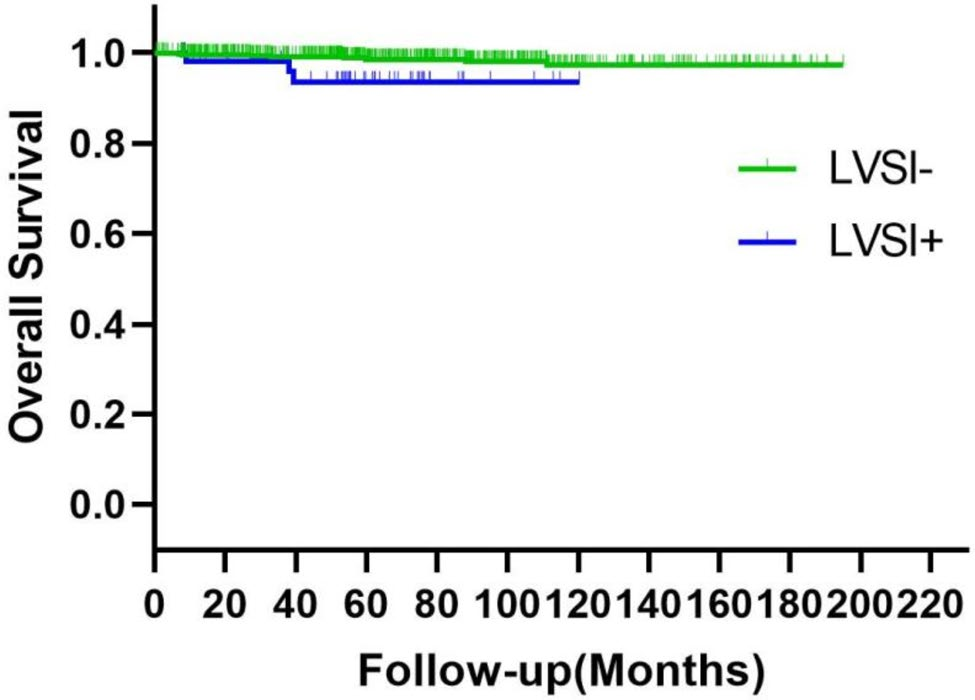
OS of patients with negative and positive LVSI. ^a^OS: overall survival; LVSI: lymphovascular space invasion

LVSI was identified as a poor prognostic factor for the risk of relapse (Fig. 2). The study found that the 5-year disease-free survival rate was 98.4% for patients who tested negative for LVSI and 93.1% for those who tested positive (*p* = 0.005). The correlation of LVSI with OS in patients was also established (Fig. 3). The 5-year overall survival rate for LVSI-negative patients was 98.9%, compared to 94.8% for LVSI-positive patients (*p* = 0.017).

We found that LVSI and Grade are two prognostic factors for EEC patients’ DFS. According to univariable Cox regression analysis, the presence of LVSI was linked to poorer DFS (HR = 4.60; 95% CI = 1.44–14.66; *p* = 0.010), as was tumor grade (HR = 5.99; 95% CI = 1.25–25.02; *p* = 0.024). On multivariable analysis, the presence of LVSI was independently related to reduced DFS (HR = 3.82; 95% CI = 1.19–12.22; *p* = 0.024). Meanwhile, tumor grade was also identified as an independent risk factor for reduced DFS (HR = 5.04; 95% CI = 1.12–22.67; *p* = 0.035) (Table 2).

**Table 2:** Univariate and multivariate analysis for diease-free survival in women with low grade and early stage endometrioid endometrial cancer

	Univariate			Multivariable	
Characteristics	HR	p		HR	p
	(95%CI)			(95%CI)	
Age					
< 60	1				
≥ 60	2.29(0.80,6.53)	0.121			
Menopausal status					
Pre-menopausal	1				
Post-menopausal	3.63(0.81,16.24)	0.091			
Grade					
G1	1			1	
G2	5.99(1.25,25.02)	0.024		5.04(1.12,22.67)	0.035
Depth of MI					
< 50%	1				
≥ 50%	1.59(0.44,5.70)	0.477			
PTD (mm), median					
≤ 20	1				
> 20	1.14(0.96,1.36)	0.139			
LVSI					
No	1			1	
Yes	4.60(1.44,14.66)	0.010		3.82(1.19,12.22)	0.024
Adjuvant					
treatment					
No	1				
Yes	2.57(0.89,7.40)	0.081			

DFS, diease free survival; HR, hazard ratio; CI, confidence interval; LVSI, lymphovascular space invasion; MI, myometrial invasion; PTD, primary tumor diameter

We also identified prognostic factors for OS (Table 3**)**. In univariable Cox regression analysis, compared with other factors, LVSI positivity was more likely to reduce OS (HR = 4.39; 95% CI = 1.17–16.49; *p* = 0.028), as was age (HR = 3.32; 95%CI = 1.05–10.48; *p* = 0.040). On multivariable analysis, LVSI presence, compared to its absence, was independently associated with worse OS (HR = 3.87; 95% CI = 1.02–14.65; *p* = 0.046) **(**Table 3**)**.

**Table 3:** Univariate and multivariable Cox regression analysis of select covariates for OS

	Univariate			Multivariable	
Characteristics	HR	p		HR	p
	(95%CI)			(95%CI)	
Age					
< 60	1			1	
≥ 60	3.32(1.05,10.48)	0.040		3.08(0.97,9.78)	0.056
Menopausal status					
Pre-menopausal	1				
Post-menopausal	3.10(0.68,14.13)	0.145			
Grading					
G1	1				
G2	0.94(0.30,2.90)	0.908			
Depth of MI					
< 50%	1				
≥ 50%	0.52(0.07,4.00)	0.526			
PTD (mm), median					
≤ 20	1				
> 20	1.13(0.95,1.35)	0.177			
LVSI					
No	1			1	
Yes	4.39(1.17,16.49)	0.028		3.87(1.02,14.65)	0.046
Adjuvant					
treatment					
No	1				
Yes	2.68(0.85,8.45)	0.093			

OS: overall survival; HR, hazard ratio; CI, confidence interval; LVSI, lymphovascular space invasion; MI, myometrial invasion; PTD, primary tumor diameter

## Discussion

This study analyzed the effect of LVSI on the prognosis of patients diagnosed with early-stage, low-grade EEC. In a cohort of 1083 EC patients, we focused on those with grade 1–2, stages IA and IB, and without other tumors. This criterion led to the selection of 702 EEC patients. After analyzing the statistics, we found (1) an LVSI prevalence of 8.3% among the 702 patients, (2) the presence of LVSI potentially impacting OS and DFS rates in early-stage and low-grade EEC patients.

The prognosis of EC is influenced by various factors, including LVSI, which is considered a significant prognostic indicator, especially in patients with early-stage and low-grade EEC [27]. The 2023 FIGO staging system acknowledges LVSI as a significant factor in assessing EC [28, 29]. Previous studies report LVSI incidences ranging from 8.9 to 13.5% in stage I EC patients [13, 18]. Our findings show that LVSI-positive patients have a lower DFS survival compared to the LVSI-negative group. Furthermore, LVSI positivity significantly affects OS in early-stage and low-grade EC patients. These findings is not conflict with our previous published cohort research, which concluded LVSI did not significantly impact EC prognosis, including all types and stages of endometrial cancer [18]. In some studies, LVSI positivity was classified into two categories: focal LVSI and diffuse LVSI. Focal LVSI indicates one or two vessels affected by the tumor found near it, while diffuse LVSI involves three or more vessels around the tumor or extensive LVSI in the myometrium with a spray-like growth pattern, irrespective of myometrial invasion severity [12]. This classification is valuable and precise, and widely utilized in clinical studies.

Except for LVSI, we identified tumor grade as another independent risk factor for the DFS rate in early stage EEC. Previous studies have not explored the correlation between the DFS rate and tumor grade in low-risk EEC [17]. After expanding the patient population, a correlation between tumor grade and DFS rate was identified. In high-grade EC, LVSI indicated statistically significant differences based on molecular classification, providing a more accurate prognosis [30]. Tumor grade is a crucial factor in early-stage and low-grade EEC prognosis.

The study found that 65.5% of LVSI-positive patients received adjuvant treatment, compared to 30.6% of LVSI-negative patients. However, adjuvant treatment showed no association with OS or DFS rates in our study. A retrospective analysis in 2023 suggested that adjuvant treatment improved the OS of LVSI-positive stage IIC EC patients, especially those with grade 3 EEC [31]. Yet, it did not influence the survival or recurrence rate in early-stage and low-grade EEC patients.

In addition, LVSI is one of the independent risk factors for lymph node metastasis in early stage endometrial cancer. Furthermore, the status of lymph nodes can affect the prognosis of patients with endometrial cancer. In recent years, the adoption of sentinel node mapping ensues a higher identification of patients with nodal disease compared with lymphadenectomy [32]. Retrospective studies have shown that the 5-year DFS rate and 5-year OS rate of patients receiving sentinel lymph node mapping are similar to those receiving lymph node dissection. Sentinel lymph node mapping has no negative impact on the 5-year prognosis of early-stage high-intermediate and high-risk endometrial cancer [33].

Further prospective evidence of the relation between sentinel node and the prognosis with endometrial cancer patients is still needed.

What’s more, our study has some limitations. Firstly, this study still needs large sample data from multiple hospital centers. Secondly, refining LVSI classification into categories such as focal and diffuse LVSI is needed. Thirdly, due to the limited number of cases with recurrence, the replase site was not stratified. Finally, the data of sentinel lymph node mapping was not included in this cohort, as this technology has been introduced to our hospital since 2015. In the future, we still need to further collect data for relevant research to confirm the objectivity and completeness of this conclusion.

## Conclusions

Overall, our findings suggest that LVSI significantly predicts DFS and OS in early-stage and low-grade EC in a fifteen-year retrospective Chinese cohort study, emphasizing its role in assessing recurrence risk. For LVSI-positive patients, prompt and adequate adjuvant treatment can effectively prevent recurrence and enhance prognosis.

## Data Availability

No datasets were generated or analysed during the current study.

## References

[1] Siegel RL, Miller KD, Wagle NS, Jemal A. Cancer statistics, 2023. CA Cancer J Clin. 2023;73(1):17–48. 36633525 10.3322/caac.21763

[2] Xia C, Dong X, Li H, Cao M, Sun D, He S, Yang F, Yan X, Zhang S, Li N, Chen W. Cancer statistics in China and United States, 2022: profiles, trends, and determinants. Chin Med J (Engl). 2022;135(5):584–90. 10.1097/CM9.0000000000002108. 35143424 10.1097/CM9.0000000000002108 PMC8920425

[3] Crosbie EJ, Kitson SJ, McAlpine JN, Mukhopadhyay A, Powell ME, Singh N. Endometrial cancer. Lancet. 2022;399(10333):1412–28. 10.1016/S0140-6736(22)00323-3. 35397864 10.1016/S0140-6736(22)00323-3

[4] Mariani A, Webb MJ, Keeney GL, et al. Low-risk corpus cancer: is lymphadenectomy or radiotherapy necessary? Am J Obstet Gynecol. 2000;182:1506–19. 10871473 10.1067/mob.2000.107335

[5] Stålberg K, Bjurberg M, Borgfeldt C, Carlson J, Dahm-Kähler P, Flöter-Rådestad A, Hellman K, Hjerpe E, Holmberg E, Kjølhede P, Marcickiewicz J, Rosenberg P, Tholander B, Åvall-Lundqvist E, Högberg T. Lymphovascular space invasion as a predictive factor for lymph node metastases and survival in endometrioid endometrial cancer - a Swedish Gynecologic Cancer Group (SweGCG) study. Acta Oncol. 2019;58(11):1628–33. Epub 2019 Aug 2. PMID: 31373248. 31373248 10.1080/0284186X.2019.1643036

[6] Li Y, Cong P, Wang P, Peng C, Liu M, Sun G. Risk factors for pelvic lymph node metastasis in endometrial cancer. Arch Gynecol Obstet. 2019;300(4):1007–1013. doi: 1.1007/s00404-019-05276-9. Epub 2019 Aug 21. PMID: 31435773. 10.1007/s00404-019-05276-9 31435773

[7] Hui C, Mendoza MG, von Eyben R, Dorigo O, Litkouhi B, Renz M, Karam A, Hammer PM, Howitt BE, Kidd E. Does lymph node assessment change the prognostic significance of substantial LVSI and p53 status in endometrial endometrioid carcinoma? Gynecol Oncol. 2023;177:150–6. Epub 2023 Sep 9. PMID: 37696217. 37696217 10.1016/j.ygyno.2023.09.001

[8] Buechi CA, Siegenthaler F, Sahli L, Papadia A, Saner FAM, Mohr S, Rau TT, Solass W, Imboden S, Mueller MD. Real-World Data assessing the impact of Lymphovascular Space Invasion on the diagnostic performance of Sentinel Lymph Node Mapping in Endometrial Cancer. Cancers (Basel). 2023;16(1):67. 10.3390/cancers16010067. PMID: 38201495; PMCID: PMC10778553. 38201495 10.3390/cancers16010067 PMC10778553

[9] Asami Y, Hiranuma K, Takayanagi D, Matsuda M, Shimada Y, Kato MK, Kuno I, Murakami N, Komatsu M, Hamamoto R, Kohno T, Sekizawa A, Matsumoto K, Kato T, Yoshida H, Shiraishi K. Predictive model for the preoperative assessment and prognostic modeling of lymph node metastasis in endometrial cancer. Sci Rep. 2022;12(1):19004. 10.1038/s41598-022-23252-3. PMID: 36347927; PMCID: PMC9643353. 36347927 10.1038/s41598-022-23252-3 PMC9643353

[10] Miller HA, Tran A, LyBarger KS, Frieboes HB. A clinical marker-based modeling framework to preoperatively predict lymph node and vascular space involvement in endometrial cancer patients. Eur J Surg Oncol. 2024;50(1):107309. 10.1016/j.ejso.2023.107309. Epub 2023 Dec 1. PMID: 38056021. 38056021 10.1016/j.ejso.2023.107309

[11] Shawn LyBarger K, Miller HA, Frieboes HB. CA125 as a predictor of endometrial cancer lymphovascular space invasion and lymph node metastasis for risk stratification in the preoperative setting. Sci Rep. 2022;12(1):19783. 10.1038/s41598-022-22026-1. PMID: 36396713; PMCID: PMC9671890. 36396713 10.1038/s41598-022-22026-1 PMC9671890

[12] Restaino S, Tortorella L, Dinoi G, Zannoni GF, Baroni A, Capasso I, Distefano E, Sozzi G, Chiantera V, Scambia G, Fanfani F. Semiquantitative evaluation of lymph-vascular space invasion in patients affected by endometrial cancer: prognostic and clinical implications. Eur J Cancer. 2021;142:29–37. Epub 2020 Nov 17. PMID: 33217679. 33217679 10.1016/j.ejca.2020.10.011

[13] Tortorella L, Restaino S, Zannoni GF, Vizzielli G, Chiantera V, Cappuccio S, Gioè A, La Fera E, Dinoi G, Angelico G, Scambia G, Fanfani F. Substantial lymph-vascular space invasion (LVSI) as predictor of distant relapse and poor prognosis in low-risk early-stage endometrial cancer. J Gynecol Oncol. 2021;32(2):e11. Epub 2021 Jan 11. PMID: 33470061; PMCID: PMC7930448. 33470061 10.3802/jgo.2021.32.e11 PMC7930448

[14] Siegenthaler F, Epstein E, Büchi CA, Gmür A, Saner FACM, Rau TT, Carlson JW, Mueller MD, Imboden S. Prognostic value of lymphovascular space invasion according to the molecular subgroups in endometrial cancer. Int J Gynecol Cancer. 2023;33(11):1702–7. 10.1136/ijgc-2023-004606. PMID: 37666529; PMCID: PMC10646877. 37666529 10.1136/ijgc-2023-004606 PMC10646877

[15] Qin ZJ, Wang YS, Chen YL, Zheng A, Han L. Evaluation of prognostic significance of lymphovascular space invasion in early stage endometrial cancer: a systematic review and meta-analysis. Front Oncol. 2024;13:1286221. 10.3389/fonc.2023.1286221. PMID: 38273843; PMCID: PMC10808564. 38273843 10.3389/fonc.2023.1286221 PMC10808564

[16] Yarandi F, Shirali E, Akhavan S, Nili F, Ramhormozian S. The impact of lymphovascular space invasion on survival in early stage low-grade endometrioid endometrial cancer. Eur J Med Res. 2023;28(1):118. 10.1186/s40001-023-01084-9. PMID: 36915143; PMCID: PMC10012545. 36915143 10.1186/s40001-023-01084-9 PMC10012545

[17] Ayhan A, Şahin H, Sari ME, Yalçin I, Haberal A, Meydanli MM. Prognostic significance of lymphovascular space invasion in low-risk endometrial cancer. Int J Gynecol Cancer. 2019;29(3):505–12. 10.1136/ijgc-2018-000069. Epub 2019 Jan 21. PMID: 30665899. 30665899 10.1136/ijgc-2018-000069

[18] Dai Y, Dong Y, Cheng Y, Hou H, Wang J, Wang Z, Wang J. Prognostic significance of lymphovascular space invasion in patients with endometrioid endometrial cancer: a retrospective study from a single center. J Gynecol Oncol. 2020;31(3):e27. 10.3802/jgo.2020.31.e27. Epub 2019 Nov 28. PMID: 31912681; PMCID: PMC7189077. 31912681 10.3802/jgo.2020.31.e27 PMC7189077

[19] Ureyen I, Karalok A, Turkmen O, Kimyon G, Akdas YR, Akyol A, Tasci T, Turan T. Factors predicting recurrence in patients with stage IA endometrioid endometrial cancer: what is the importance of LVSI? Arch Gynecol Obstet. 2020;301(3):737–44. 10.1007/s00404-019-05418-z. Epub 2019 Dec 27. PMID: 31883046. 31883046 10.1007/s00404-019-05418-z

[20] Pifer PM, Jaishankar S, Bhargava R, Schad MD, Keller A, Musunuru HB, Cohen M, Sukumvanich P, Courtney-Brooks M, Boisen M, Berger JL, Olawaiye A, Lesnock J, Edwards R, Taylor S, Vargo JA, Beriwal S. Is substantial Lymphovascular Space Invasion Prognostic in patients with pathologically Lymph Node-negative endometrial Cancer? Int J Radiat Oncol Biol Phys 2023 Mar 8:S0360-3016(23)00214-6. 10.1016/j.ijrobp.2023.02.053. Epub ahead of print. PMID: 36893818. 10.1016/j.ijrobp.2023.02.053 PMC11225593 36893818

[21] Navarro B, Margioula-Siarkou C, Petousis S, Floquet A, Babin G, Guyon F. Surgical restaging of patients with early–stage endometrial cancer with lymphovascular invasion does not significantly impact their survival outcomes. Oncol Lett. 2023;25(3):122. 10.3892/ol.2023.13708. PMID: 36844624; PMCID: PMC9950339. 36844624 10.3892/ol.2023.13708 PMC9950339

[22] Chen KS, Berhane H, Gill BS, Olawaiye A, Sukumvanich P, Kelley JL, Boisen MM, Courtney-Brooks M, Comerci JT, Edwards R, Berger J, Beriwal S. Outcomes of stage II endometrial cancer: the UPMC Hillman Cancer Center experience. Gynecol Oncol. 2017;147(2):315–9. Epub 2017 Aug 31. PMID: 28866431. 28866431 10.1016/j.ygyno.2017.08.021

[23] Zheng W. Molecular classification of Endometrial Cancer and the 2023 FIGO staging: exploring the challenges and opportunities for pathologists. Cancers (Basel). 2023;15(16):4101. 10.3390/cancers15164101. PMID: 37627129; PMCID: PMC10452831. 37627129 10.3390/cancers15164101 PMC10452831

[24] Dou Y, Song K, Fu Y, Shen Y, Zhang C, Yao S, Xu C, Xia M, Lou G, Liu J, Lin B, Wang J, Zhao W, Zhang J, Cheng W, Guo H, Guo R, Xue F, Wang X, Han L, Zhao X, Li X, Zhang P, Zhao J, Ma J, Li W, Yang X, Wang Z, Liu J, Fang Y, Li K, Chen G, Sun C, Cheng X, Jiang J, Wang B, Luo D, Kong B. Chinese Endometrial Carcinoma Consortium (CECC). Risk factors and prognosis of early recurrence in Stage I-II Endometrial Cancer: a Large-Scale, Multi-center, and Retrospective Study. Front Med (Lausanne). 2022;9:808037. 10.3389/fmed.2022.808037. PMID: 35492356; PMCID: PMC9046937. 35492356 10.3389/fmed.2022.808037 PMC9046937

[25] Garzon S, Grassi T, Mariani A, Kollikonda S, Weaver AL, McGree ME, Petersen IA, Weroha SJ, Glaser GE, Langstraat CL, Amarnath SR, AlHilli MM. Not all stage I and II endometrial cancers are created equal: recurrence-free survival and cause-specific survival after observation or vaginal brachytherapy alone in all subgroups of early-stage high-intermediate and high-risk endometrial cancer. Gynecol Oncol. 2022;167(3):444–51. 10.1016/j.ygyno.2022.10.004. Epub 2022 Oct 14. PMID: 36244826. 36244826 10.1016/j.ygyno.2022.10.004

[26] Creasman WT, Odicino F, Maisonneuve P, Quinn MA, Beller U, Benedet JL, Heintz AP, Ngan HY, Pecorelli S. Carcinoma of the corpus uteri. FIGO 26th Annual Report on the Results of Treatment in Gynecological Cancer. Int J Gynaecol Obstet. 2006;95 Suppl 1:S105-43. 10.1016/S0020-7292(06)60031-3. PMID: 17161155. 10.1016/S0020-7292(06)60031-3 17161155

[27] Keys HM, Roberts JA, Brunetto VL, Zaino RJ, Spirtos NM, Bloss JD, Pearlman A, Maiman MA, Bell JG, Gynecologic Oncology Group. A phase III trial of surgery with or without adjunctive external pelvic radiation therapy in intermediate risk endometrial adenocarcinoma: a Gynecologic Oncology Group study. Gynecol Oncol. 2004;92(3):744–51. 10.1016/j.ygyno.2003.11.048. Erratum in: Gynecol Oncol. 2004;94(1):241-2. PMID: 14984936. 14984936 10.1016/j.ygyno.2003.11.048

[28] Berek JS, Matias-Guiu X, Creutzberg C, Fotopoulou C, Gaffney D, Kehoe S, Lindemann K, Mutch D, Concin N. Endometrial Cancer staging Subcommittee, FIGO Women’s Cancer Committee. FIGO staging of endometrial cancer: 2023. J Gynecol Oncol. 2023;34(5):e85. 10.3802/jgo.2023.34.e85. Epub 2023 Aug 8. PMID: 37593813; PMCID: PMC10482588. 37593813 10.3802/jgo.2023.34.e85 PMC10482588

[29] Berek JS, Matias-Guiu X, Creutzberg C, Fotopoulou C, Gaffney D, Kehoe S, Lindemann K, Mutch D, Concin N. Endometrial Cancer Staging Subcommittee, FIGO Women’s Cancer Committee. FIGO staging of endometrial cancer: 2023. Int J Gynaecol Obstet. 2023;162(2):383–394. 10.1002/ijgo.14923. Epub 2023 Jun 20. Erratum in: Int J Gynaecol Obstet. 2023;: PMID: 37337978. 10.1002/ijgo.14923 37337978

[30] Bayramoglu D, Seçilmiş Kerimoğlu Ö, Bayramoğlu Z, Çintesun E, Şahin G, Karabağlı P, Çelik Ç. Classification of high-grade endometrium carcinomas using molecular and immunohistochemical methods. Ginekol Pol. 2023;94(1):3–11. 10.5603/GP.a2021.0177. Epub 2022 Jan 24. PMID: 35072228. 35072228 10.5603/GP.a2021.0177

[31] Lee YY, Lai YL, Kim MS, Chang K, Kim HS, Cheng WF, Chen YL. Impact of adjuvant treatment on survival in patients with 2023 FIGO stage IIC endometrial cancer: a retrospective analysis from two tertiary centers in Korea and Taiwan. J Gynecol Oncol. 2023 Dec 12. 10.3802/jgo.2024.35.e33. Epub ahead of print. PMID: 38130137. 10.3802/jgo.2024.35.e33 PMC11107281 38130137

[32] Bogani G, Giannini A, Vizza E, Di Donato V, Raspagliesi F. Sentinel node mapping in endometrial cancer. J Gynecol Oncol. 2024;35(1):e29. 10.3802/jgo.2024.35.e29. Epub 2023 Nov 13. PMID: 37973163; PMCID: PMC10792208. 37973163 10.3802/jgo.2024.35.e29 PMC10792208

[33] Cuccu I, Raspagliesi F, Malzoni M, Vizza E, Papadia A, Di Donato V, Giannini A, De Iaco P, Perrone AM, Plotti F, Angioli R, Casarin J, Ghezzi F, Cianci S, Vizzielli G, Restaino S, Petrillo M, Sorbi F, Multinu F, Schivardi G, De Vitis LA, Falcone F, Lalli L, Berretta R, Mueller MD, Tozzi R, Chiantera V, Benedetti Panici P, Fanfani F, Scambia G, Bogani G. Sentinel node mapping in high-intermediate and high-risk endometrial cancer: analysis of 5-year oncologic outcomes. Eur J Surg Oncol. 2024;50(4):108018. 10.1016/j.ejso.2024.108018. Epub 2024 Feb 15. PMID: 38428106. 38428106 10.1016/j.ejso.2024.108018

